# COVID-19 Stress and Family Well-Being: The Role of Sleep in Mental Health Outcomes for Parents and Children

**DOI:** 10.3390/children12080962

**Published:** 2025-07-22

**Authors:** Alzena Ilie, Andy J. Kim, Danika DesRoches, Elizabeth Keys, Simon B. Sherry, Sherry H. Stewart, S. Hélène Deacon, Penny V. Corkum

**Affiliations:** 1 Department of Psychology and Neuroscience, Faculty of Science, Dalhousie University, Halifax, NS B3H 4R2, Canada; alzenailie@dal.ca (A.I.);; 2 School of Nursing, University of British Columbia Okanagan, Kelowna, BC V1V 1V7, Canada; 3 Department of Psychiatry, Faculty of Medicine, Dalhousie University, Halifax, NS B3K 6R8, Canada

**Keywords:** COVID-19 stress, mental health, sleep, well-being, family

## Abstract

Background/Objectives: The COVID-19 pandemic introduced various stressors for families, including changes to daily routines, work, and schooling. Studies have linked these stressors to increased mental health challenges for parents and children. Sleep difficulties were also common during the pandemic, with some children and parents experiencing poorer sleep quality and shorter sleep duration. However, it remains unclear whether the effects of COVID-19 stress on mental health challenges are explained, at least in part, by effects of COVID-19 stress on child and/or parent sleep challenges. This study examined the impacts of COVID-19 stress on sleep and, in turn, mental health difficulties in school-aged children and their parents in Canada and the United States. Methods: Parents (*N* = 961) completed validated measures of COVID-19 stress, and of their own and their child’s sleep and mental health. Path analyses tested direct and indirect effects of COVID-19 stress on mental health outcomes with sleep problems as the potential mediator. Results: Child sleep problems partially mediated COVID-19 stress effects on both parent (β = 0.33) and child (β = 0.20) mental health difficulties, while parent sleep problems contributed significantly but to a lesser degree (parent mental health: β = 0.07; child mental health: β = 0.03). There also remained significant direct effects of COVID-19 stress on both child and parent mental health difficulties that were not mediated through sleep difficulties. Conclusions: Our findings underscore the interconnected nature of sleep and mental health, demonstrating that stress-related disruptions in sleep (particularly children’s sleep) can exacerbate mental health difficulties for both parents and children during crises like the COVID-19 pandemic.

## 1. Introduction

Throughout the world, public health recommendations related to the COVID-19 pandemic resulted in unprecedented changes to everyday life [1,2]. These changes aimed to urgently reduce the spread of the virus. Yet, they also led many families with school-aged children in Canada and the United States to experience significant disruptions to their routines [3]. For example, the closure of in-person schooling, the transition to remote learning, and closure of organized activities (e.g., sports, music lessons, organized social activities) significantly changed some children’s routines, which impacted their learning, socialization, and extracurricular activities [3].

School-aged children are a population particularly vulnerable to the effects of disruptions in routine due to their developmental need for structure, consistency, and predictable daily schedules, which support both academic and social development [4]. Research underscores the importance of consistent daily routines, which are closely linked to positive child development and mental health outcomes [4]. Stability in routines provides a sense of predictability and security that supports emotional regulation and overall well-being in children. However, the pandemic disrupted this stability for many families, leading to varied outcomes. While some families found these disruptions to be positive, enabling more family time and a slower pace of life, others reported negative impacts such as heightened stress, problematic behaviours, and emotional challenges in both children and parents [5,6]. For instance, caregivers noted increases in children’s anhedonia, anger, restlessness, sleep problems, appetite issues, and sadness after the COVID-19 pandemic started [3,7]. These behavioural and emotional challenges are critical indicators of broader mental health difficulties, as they may reflect disruptions in coping mechanisms, emotional dysregulation, and difficulties with overall psychological well-being. Observed behavioural changes, such as increased sedentary activities, later sleep timing, and heightened screen use, underscore the impact of the pandemic on daily routines [8]. During the COVID-19 pandemic, declines in physical activity, increases in screen time, later bedtimes, and unhealthy eating behaviours were reported in Canadian children [9]. These lifestyle disruptions may adversely interact with stress and sleep behaviours, compounding difficulties in maintaining healthy routines under pandemic conditions [10].

Insomnia and/or insomnia symptoms affect ~30% of typically developing children [11], and include difficulties with falling asleep, staying asleep, and/or having early morning awakenings [12]. Sleep has a reciprocal role in the mental well-being of children: after children have a good night’s sleep, they tend to report higher positive affect and lower negative affect throughout the day [13]. In contrast, insufficient quantity and poor-quality sleep are associated with worse mood and emotion regulation in youth, as well as increased likelihood of developing a mood or anxiety disorder, and heightened risk of suicidal ideation [14]. Sleep duration and latency have been strongly correlated with internalizing (e.g., depression, anxiety) and externalizing (e.g., aggression, hyperactivity) behaviours in children [15].

The cyclical relationship between sleep and mental health is well established: poor sleep exacerbates mental health difficulties, while mental health challenges disrupt sleep [15]. The correlation between sleep and mental health difficulties becomes even more heightened under conditions of stress [16,17], such as COVID-19-specific stressors. For example, Mackenzie and colleagues found that disruptions to routines during the pandemic adversely affected the sleep and stress levels of parents and children with pre-existing insomnia [6]. Indeed, although some families reported improved sleep, many parents reported sleep disruptions for both themselves and their children: some 40% of parents indicated their child’s sleep worsened, while 60% reported declines in their own sleep quality [6]. Similarly, Cellini and colleagues demonstrated that the majority of children experienced delayed sleep timing (e.g., later bedtime and wakeup times), worsened sleep quality, and increased psychological difficulties during the pandemic [18]. Cellini et al. found that these child sleep and mental health difficulties were associated with maternal psychological challenges, suggesting a reciprocal relationship between caregiver and child sleep and well-being [18]. COVID-19 stress has had significant impacts on parent and child mental health outside of North America, such as in Norway and Greece [19,20].

Poor mental health in children has been a persistent issue prior to the COVID-19 pandemic. In Canada, approximately one in five children and youth have a diagnosable mental health condition [21]. These challenges are associated with lower academic achievement, higher stress levels, increased risk of substance use, and diminished social support [22]. During the pandemic, mental health problems in children and youth worsened [23,24,25], likely exacerbated by heightened stressors and disrupted routines. Cost and colleagues surveyed school-aged children and their caregivers in Canada during the COVID-19 pandemic and found that over two-thirds of children experienced worsened mental health symptoms, which were linked to stress related to social isolation [23]. Similarly, parental mental health problems, which increased during the pandemic, were strongly associated with worse mental health outcomes in children [26]. For example, Canadian parents with children reported higher rates of stress, alcohol consumption, suicidal ideation, and negative parent–child interactions during the pandemic [5]. These findings underscore the cyclical nature of the stress–sleep–mental health relationship [27], wherein stress exacerbates sleep difficulties and mental health challenges, which in turn amplify stress.

Despite this knowledge, there are key remaining questions as to the specific relations between stress, sleep, and mental health in children in the context of COVID-19. Given the unprecedented and prolonged disruptions caused by the pandemic, including school closures, social isolation, and disruptions to daily routines, stress levels in children and parents were found to be amplified during the pandemic compared to typical stressors [3]. Evidence from pre-pandemic research suggests that sleep may mediate the relationship between stress and mental health, with sufficient sleep mitigating the adverse effects of stress, and poor sleep exacerbating these adverse effects [16,17]. For example, children with sufficient sleep report less negative affect and better emotion regulation following a stressful situation [28]. Conversely, insufficient sleep heightens the risk of emotional dysregulation and psychological disorders after experiencing a stressful event [28]. Since sleep is a more modifiable variable than broader pandemic-related stressors, identifying sleep as a key mechanism could inform targeted strategies to support child mental health through pandemics and other events that have broad societal impacts.

Understanding these relationships is particularly important, as sleep disruptions may contribute to reciprocal and cross-over effects within families [29], beyond their likely mediation in the link between COVID-19 stress and mental health difficulties. For instance, COVID-19 stress could worsen child sleep disruptions, and in turn worsen parental mental health, while parental sleep difficulties may contribute to dysregulation in children [5,6]. Previous research highlights associations between parent and child sleep and mental health, suggesting that disruptions in one family member’s sleep can have cascading effects on the entire household [30]. Thus, studying both parent and child sleep within the context of pandemic-related stress is critical for capturing the full scope of its impact on family mental health. This is a novel contribution of our study design, as no studies to date have examined the relations of COVID-19-related stressors, sleep, and mental health in a sample of both parents and children.

The objectives of this study were to (a) describe sleep behaviours and mental health symptoms during the COVID-19 pandemic in school-aged children (aged 5 to 11 years) and their parents, (b) test sleep disruption as a mediator between COVID-19 stress and mental health outcomes, and (c) examine the relative mediating roles of parental and child sleep disruptions in predicting both parent and child mental health outcomes. By exploring these relationships, this study aimed to provide a deeper understanding of how stress, sleep, and mental health intersected during the COVID-19 pandemic.

This paper reports on secondary analysis of data from a larger study examining the effects of COVID-19 pandemic mandated homeschooling on families entitled *COVID-19 Pandemic: Factors that Support and Impede Family Well-Being During Mandatory Homeschooling* conducted by the Language and Literacy Lab and the Mood, Anxiety and Co-Morbidity Lab at Dalhousie University [31,32,33]. This study included children engaging in one of three types of schooling (i.e., in-person learning, mandated homeschooling, and voluntary homeschooling).

## 2. Materials and Methods

### 2.1. Study Design and Sample

A total of 961 parents were recruited from March to May 2021 for this cross-sectional study. To be eligible for the larger study, participants were required to be (a) at least 19 years of age, (b) in a romantic relationship for 3 months or more, (c) with a partner who is at least 19 years of age, and (d) have a child at home in grades 1 to 5. These criteria ensured that only couples who were romantically involved for more than three months and who lived with a school-aged child participated in the larger study, as it looked at the effects of homeschooling on parental, child and couple-level variables. While couples were recruited for the larger study, only data from the first responding parent (who reported on themselves and their child) was utilized in the present study. When a parent had more than one child in grades 1 to 5 living at home, they reported on the youngest eligible child. No other inclusion or exclusion criteria were employed for our specific study.

### 2.2. Procedures

Participants completed a survey via Qualtrics Survey Panels (Qualtrics, Provo, UT, USA) between March and May 2021. Qualtrics Survey Panels is an online survey management service that recruits from a pool of potential participants based on eligibility criteria specified by the researcher. The participant was asked to think about the 30-day period between 15 January and 15 February 2021 as the reference for completing all measures except for demographics. Although the survey was open to all parents in the general population living in Canada and the United States, recruitment was targeted in specific geographical areas where in-person learning was occurring (i.e., Canada: Edmonton, Calgary, Vancouver, Halifax, Montreal; USA: Miami and Jacksonville) and where mandated homeschooling (i.e., children who learned at home due to reasons related to the COVID-19 pandemic) was in effect (i.e., Canada: Toronto; USA: Los Angeles, Philadelphia, San Diego, Boston) at the same time (from 15 January to 15 February 2021) [34].

About two-thirds of participants were recruited and compensated through Qualtrics Panels using their guidelines. Additional recruitment was needed, for reasons related to the parent study, of parents who schooled a child at home voluntarily irrespective of COVID-19 restrictive measures, due to low recruitment via Qualtrics Panels. For these additional participants, recruitment was performed via social media platforms and homeschooling groups, and these participants were compensated with CAD 10 Amazon e-gift cards. This study was approved by the Dalhousie University Social Sciences and Humanities Research Ethics Board (REB #2020-5336).

### 2.3. Measures

#### 2.3.1. Demographics

The demographics questionnaire asked the respondent to self-report their age, gender, highest level of education completed, employment status, family income, and location (i.e., province or state). The parent also reported on their child’s age, gender, ethnicity, and the number of children living in the home.

#### 2.3.2. Pandemic-Related Stress

Parent COVID-19 stress. The presence of COVID-19 stressors was measured using an adapted version of the Coronavirus Stressor Survey [35]. The Coronavirus Stressor Survey [35] asks participants to rate their exposure to the following seven pandemic-related stressful experiences: “job requires possible exposure to coronavirus”, “lost job, been laid off or furloughed, due to the coronavirus pandemic”, “lost income due to the coronavirus pandemic”, “difficulty getting food, medication, or other necessities due to the coronavirus pandemic”, “had a loved one die due to confirmed or suspected coronavirus infection”, “work has moved to work-from-home due to coronavirus pandemic”, and “difficulty getting needed social support due to the coronavirus pandemic”. The only adaptation to the list of COVID-19 stressors was that we also asked participants to indicate whether they have had/have COVID-19 [35].

Response options were adapted to allow participants to rate to what extent they have been affected by the seven pandemic-related stressful experiences on a scale of 0 (“it did not happen to me”) to 6 (“affected me to a great extent”) since the beginning of the pandemic. Consistent with past research [36,37], and because we were interested in exposure severity, we re-coded responses to indicate whether each stressful experience had or had not happened to them (0—no, it did not happen to the participant; 1—yes, it did happen to the participant). Anytime the participant rated an experience as anything other than 0, the response was recoded as a 1, regardless of severity. A total score ranging from 0 to 8 was computed by summing all eight items. The measure showed good internal consistency (α = 0.85).

#### 2.3.3. Child and Parent Mental Health

Child mental health. Child mental health was assessed using the internalizing and externalizing scales of the Strengths and Difficulties Questionnaire (SDQ) [38]. The parent indicated how true each item was of their child on a 3-point scale (0 = “Not true”; 1 = “Somewhat true”; 2 = “Certainly true”). Twenty items asked about internalizing problems, half of which assessed emotional problems (e.g., “Often unhappy, depressed or tearful”), and the other half of which assessed peer problems (e.g., “Has at least one good friend” [reverse scored]). Another twenty items asked about externalizing problems, half of which measured conduct problems (e.g., “Often fights with other children or bullies them”) and the other half of which measured hyperactivity problems (e.g., “Restless, overactive, cannot stay still for long”). A total SDQ score, representing overall child mental health symptoms, was calculated by summing responses from both the internalizing and externalizing scales and could range from 0 to 40 following reverse scoring of any inversely keyed items. The SDQ has been standardized for 4- to 16-year-olds, and has shown both good convergent and discriminant validity with other child mental health scales [38] and internal consistency [39]. Similarly, the current sample demonstrated good internal consistency for the SDQ total (α = 0.83).

Parent mental health. Participants self-reported on their own mental health by completing both the Patient Health Questionnaire-9 (PHQ-9) [40], a measure of depressive symptoms, and the Generalized Anxiety Disorder-7 (GAD-7) questionnaire [41], a measure of anxiety symptoms. Participants rated nine items on the PHQ-9 and seven items on the GAD-7. Both scales were on a 4-point Likert scale ranging from 0 (not at all) to 3 (nearly every day). Both the PHQ-9 (α = 0.89) [40] and the GAD-7 (α = 0.92) [41] have shown good internal consistency in past research.

A composite mental health score, known as the Anxiety–Depression Scale (ADS), was computed for the responding parent [42]. Previous research on the ADS has shown high internal reliability in three clinical trials (α ranging from 0.80 to 0.90) [42]. Two of the nine items of the PHQ-9 (i.e., Items 3 and 4) were dropped due to their redundancy with the parent sleep measure used in this study. As such, a modified ADS total score was computed by summing the remaining items of the PHQ-9 and the GAD-7, with higher scores indicating worse mental health through higher levels of depression and anxiety symptomology. The modified ADS demonstrated excellent internal reliability in this study (α = 0.95).

#### 2.3.4. Sleep

Child sleep. Child sleep problems were measured using the Patient-Reported Outcomes Measurement Information System (PROMIS) Parent Proxy Short Form v1.0—Sleep Disturbance—4a [43]. Participants rated four statements, such as “My child had difficulty falling asleep” on a scale from 1 (never) to 5 (always). The four items were then summed to compute a total score, ranging from 4 to 20, with a higher score indicating worse sleep. Research has indicated that this measure has excellent psychometric properties, including internal consistency (α > 0.90) and content, structural and convergent validity with other child sleep and fatigue measures [44]. This measure had acceptable internal consistency within our own study (α = 0.75).

Parent sleep. Parent sleep difficulties were assessed using the PROMIS Short Form v1.0—Sleep Disturbance—4a [43]. Participants self-reported on their own sleep by rating four statements on a 5-point Likert scale (e.g., “I had a problem with my sleep”), which were summed together to create a final score ranging from 4 to 20 (higher score indicating worse sleep). This scale has shown good convergent and discriminant validity with other adult sleep measures [45]. In our study, this measure showed acceptable internal consistency (α = 0.77), falling in line with previous research (α = 0.88) [45].

### 2.4. Data Analysis

Descriptive statistics, including means and standard deviations for continuous variables and percentages for categorical variables, were calculated for the demographic questionnaire. Bivariate correlations were conducted to examine the relationships among the predictor, mediator, and outcome variables. Correlation strength was interpreted using the following guidelines: a small correlation was defined as an *r*-value between 0.10 and 0.29, a moderate correlation was an *r*-value between 0.30 and 0.49, and a strong correlation was an *r*-value of 0.50 or higher [46]. A path model was fitted, with COVID-19 stress as the predictor, child and parent sleep problems as mediators, and child and parent mental health as outcomes, using R (version 4.2.1) with the *lavaan* package [47]. Given that parent and child scores were reported by the parent, we accounted for their interdependence by specifying correlations between the errors of child and parent sleep problems, as well as between child and parent mental health difficulties. To estimate the confidence intervals for all coefficients and the significance of indirect effects, bias-corrected standard errors were computed using bootstrapping with 5000 re-samples [48]. The model was just identified, as all paths were required to estimate total effects and mediation effect sizes. Consequently, there are no fit indices to report.

## 3. Results

The parent and child demographic information is reported in Table 1 and Table 2. Overall, parents’ mean age was 38.21 years old, with an almost equal distribution of male and female participants. Most parents identified as White, were college/university graduates, were employed, had a family income of at least CAD 76,000 per year, and lived in Central Canada (i.e., Ontario and Quebec). The mean age of the child on whom participants were reporting was 8.64 years old. In terms of gender, the majority (57%) of children were male, with the majority reported to be White.

Table 3 presents bivariate correlations and descriptive statistics. The bivariate correlations indicated moderate positive correlations between COVID-19 stress and child sleep problems, as well as between COVID-19 stress and parent mental health difficulties. There was a strong positive correlation between COVID-19 stress and child mental health difficulties, whereas a small correlation was found between COVID-19 stress and parent sleep problems. Child sleep problems showed strong positive correlations with both child and parent mental health difficulties, which were also strongly correlated with one another. Similarly, parent sleep problems were strongly and positively correlated with parent mental health difficulties and moderately correlated with child mental health difficulties and sleep problems.

**Figure 1:**
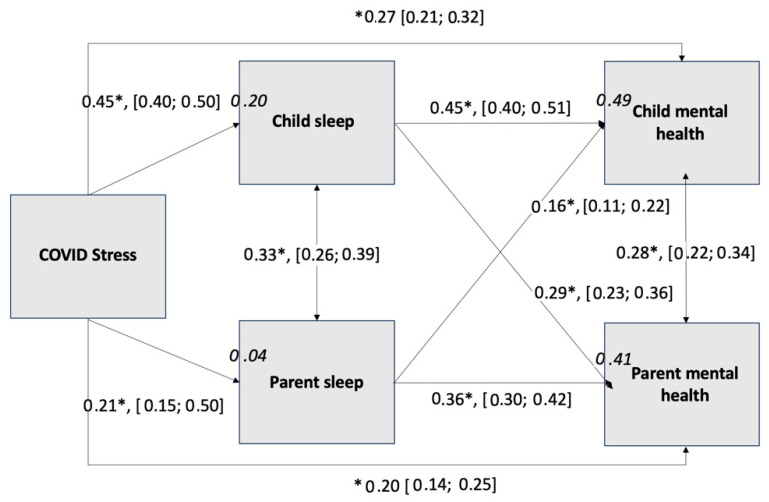
Path analysis of COVID-19 stress, sleep problems, and mental health. *Note. N* = 961. Double-headed arrows indicate correlations between error terms. Single-headed arrows indicate paths. All paths noted with asterisk are significant (*p* < 0.001). Path coefficients are standardized. Bracketed values indicate the lower and upper limits of confidence intervals. Italicized numbers appearing in the bubbles represent the R^2^.

The path model (Figure 1) tested the hypothesized relationships between COVID-19 stress (predictor) and both child mental health and parent mental health difficulties (outcomes)—both direct effects and indirect effects through child and parent sleep problems (mediators).

Significant indirect effects of COVID-19 stress on child mental health difficulties were observed through child sleep problems (β = 0.20, 95% CI [0.17; 0.24]) and, to a lesser extent, through parent sleep problems (β = 0.03, 95% CI [0.02; 0.05]). This suggests that for every 1 standard deviation increase in COVID-19 stress, child mental health difficulties increase by 0.20 and 0.03 standard deviations through child and parent sleep problems, respectively.

The mediation analysis also revealed significant indirect effects of COVID-19 stress on parent mental health difficulties through child sleep problems (β = 0.13, 95% CI [0.10; 0.16]) and through parent sleep problems (β = 0.07, 95% CI [0.05; 0.10]). Specifically, for every 1 standard deviation increase in COVID-19 stress, parent mental health difficulties increase by 0.13 and 0.07 standard deviations through child and parent sleep problems, respectively.

In terms of direct effects, the path coefficients indicated significant relationships between COVID-19 stress and both child and parent mental health difficulties. Additionally, the path coefficients indicated significant relationships between COVID-19 stress and both child and parent sleep problems. Significant relationships were also observed in the direct paths from child sleep problems to child mental health difficulties, from parent sleep problems to parent mental health difficulties, from child sleep problems to parent mental health difficulties, and from parent sleep problems to child mental health difficulties. The error variances for child and parent sleep problems were moderately correlated with one another, as were the error variances for child and parent mental health difficulties.

We examined the 95% CIs to determine which indirect effects were significantly stronger than other mediational pathways; a given set of mediational pathways was considered significantly different from one another if the 95% CIs were not overlapping. Child sleep problems was a stronger mediator than parent sleep problems in explaining the link between COVID-19 stress and child mental health difficulties; in contrast child and parent sleep problems were similar in magnitude as mediators in explaining the link between COVID-19 stress and parent mental health difficulties. Additionally, child sleep problems was a stronger mediator of the link between COVID-19 stress and mental health difficulties in the case of child mental health difficulties than in the case of parent mental health difficulties; in contrast, parent sleep problems showed a similar magnitude mediational effect in the link between COVID-19 stress and mental health difficulties for both child and parent.

The total effects of COVID-19 stress on parent mental health (β = 0.40, 95% CI [0.34; 0.46]) and child mental health (β = 0.51, 95% CI [0.46; 0.55]) were computed by summing their associated direct and indirect effects.

The percent mediation is the percentage of a total effect that is accounted for by indirect effect(s). We first calculated the percent mediation for parent mental health difficulties by dividing the sum of both its indirect effects by its total effect (51%). We did the same for child mental health difficulties (47%). This percentage was further broken down for parent mental health difficulties into the percent mediated by the individual indirect effects of parent sleep problems (18% of the 51%) and child sleep problems (33% of the 51%), as well as parent sleep problems (7% of the 47%) and child sleep problems (40% of the 47%) for child mental health difficulties.

## 4. Discussion

To our knowledge, this study is the first to examine the relationship between COVID-19 stress, parent and child mental health, and sleep behaviours in both children and parents, filling an important gap in the literature, and potentially providing intervention targets during times of high stress. In this large cohort of 961 parent and child dyads from Canada and the US who had experienced varying levels of COVID-19 related stress, we conducted a secondary analysis to determine the role of sleep in mediating the relationship between COVID-19 stress and mental health outcomes in parents and children. Overall, the path analysis found significant indirect effects between COVID-19 stress, sleep problems in children and parents, and their contributions to mental health difficulties. Additionally, there were significant direct effects of COVID-19 stress on parent and children mental health difficulties.

The path analysis revealed significant indirect effects: COVID-19 stress was linked to sleep problems in both children and parents, and these sleep problems, in turn, contributed to mental health difficulties. Specifically, both child and parent sleep were partial mediators of the effect of COVID-19 stress on mental health. For children, their own sleep was a stronger predictor of their mental health outcomes, while for parents, both their own sleep and their child’s sleep were significant predictors. These findings suggest that sleep may serve as a key pathway through which external stressors impact psychological well-being across family members [51,52].

In the mediation analysis, COVID-19 stress indirectly affected parents’ mental health through both their own sleep problems and their child’s sleep problems. For children, COVID-19 stress indirectly impacted their mental health through their own sleep problems and their parent’s sleep problems. While COVID-19 stress had direct effects on both parent and child mental health difficulties, a significant portion of these effects was mediated by sleep problems. Child sleep problems accounted for a larger portion of the mediation on parent mental health compared to parent sleep problems. Moreover, child sleep problems played a significantly stronger role in mediating the link between COVID-19 stress and parent mental health difficulties compared to parent sleep problems. Our findings are consistent with previous research that has shown when children have sleep difficulties, it can cause insomnia symptoms and higher pre-sleep arousal in parents [51], and that child sleep mediates the association between parent’s sleep and mood. Additionally, another study found that children’s sleep problems were significant predictors of sleep problems in parents, and subjective reports of children’s sleep schedules impacted parents’ mental health [52]. This demonstrates that disruption in children’s sleep can increase both child behavioural difficulties and risk of parental burnout [53,54]. Therefore, the current study suggests that the association between COVID-19 stress and child mental health problems appear to be partially explained by the link between child sleep and mental health.

Interestingly, child sleep problems had a larger mediation effect on child mental health difficulties compared to parent sleep problems and their mediational effect on parent mental health. This may be because, according to Canadian and American sleep guidelines [55,56], children require more hours of sleep per night than adults do, and therefore, adults may be able to function better with less sleep. Furthermore, child sleep is directly linked to brain development across childhood and adolescence, mood, emotional regulation, and academic achievement, which further highlights the importance of children getting the recommended hours of sleep per night [57]. Aligning with our results, a meta-analysis by Bussières and colleagues found that the COVID-19 pandemic had a negative influence on children’s mental health, with anxiety and depression symptoms being two times higher than pre-pandemic estimates [58]. In our results, parent sleep problems showed a small magnitude correlation with COVID-19 stress, though parent mental health difficulties were moderately correlated with child sleep problems, highlighting a broader interrelation between sleep and mental health outcomes. Previous research has demonstrated that child sleep problems impact parents’ sleep by reducing their total sleep time [51], in turn, negatively impacting parents daytime functioning and mood [53,59]. This may explain why child sleep problems proved stronger mediators than parent sleep problems when examining the effect of COVID-19 stress on both child and parent mental health difficulties.

Consistent with the supporting literature [58,60,61], the path analysis found that COVID-19 stress directly impacted parent and child mental health, even after accounting for sleep problems. This suggests that COVID-19 stress may influence mental health through other factors, such as financial strain, increased caregiver burden, and disruption to routines. In addition, the presence of direct effects reinforces the robustness of the relationship between pandemic-related stressors and mental distress in children and parents, aligning with previous research on the impact of the COVID-19 pandemic on increased internalizing symptoms in both parents and children [5,23,24,25,26,27].

When examining the confidence intervals of the indirect effects, child sleep problems were a significantly stronger mediator of the relationship between COVID-19 stress and child mental health difficulties compared to parent sleep problems. This finding highlights the particularly important role of child sleep in shaping children’s emotional and behavioural responses to stress. In contrast, child and parent sleep problems were comparable in their mediating roles when explaining the link between COVID-19 stress and parent mental health difficulties, suggesting that both self- and child-related sleep disruptions contribute meaningfully to parents’ psychological well-being. Taken together, these results underscore the differential impact of sleep disruptions depending on whether the mental health outcomes pertain to the child or the parent. These findings also reinforce the need to consider both sleep and family dynamics when supporting mental health during periods of high stress.

When combining direct and indirect effects, COVID-19 stress had a large overall impact on both child and parent mental health difficulties. The strength of these associations suggests that COVID-19 stress functioned as a risk factor for family mental health difficulties, consistent with broader literature documenting heightened rates of depression, anxiety, and distress in both children and adults during the pandemic [58,62].

### 4.1. Strengths

The large sample size (*N* = 961) and relatively equal distribution between mothers (*n* = 478) and fathers (*n* = 483) in the sample is a strength, as it increased statistical power and enhanced the reliability of the findings. Path analysis was also used, which allowed for the examination of both direct effects of COVID-19 stress on child and parent mental health and indirect effects through sleep, providing a nuanced understanding of how COVID-19 stress impacts mental health in both children and parents. In addition, the current study sample was racially diverse, and was roughly representative of the Canadian and American population, with ~70% participants identifying as White, and ~30% of participants identifying as part of a non-majority racial/ethnic background [63,64]. The high R^2^ values observed in the study indicate that a substantial portion of the variance in mental health outcomes was explained by the model regarding the impact of COVID-19 stress on both parent and child mental health. This suggests that the findings have a strong explanatory power in understanding how stressors during the pandemic affected child and parent well-being.

### 4.2. Limitations

The analyses included in this study were cross-sectional, so temporal precedence and causality cannot be determined between variables. A longitudinal analysis could have provided insight into the persistence of COVID-19 stress on sleep and mental health beyond the immediate pandemic context. While some researchers argue that mediation analyses should not be conducted on cross-sectional data since mediation is a process that unfolds over time [65], others suggest that such analyses can serve as a useful starting point for testing novel models, such as the path model examined in this study [66].

Due to the design of the larger study from which the data were drawn [31,32,33], all parents who participated in this study were in a romantic relationship and were co-parenting. This may have influenced the study’s results and may limit our ability to generalize the findings to single-parent households. Given that single-parent households often have reduced social and parenting support [67], the effects of COVID-19 stress on parent and child sleep and mental health may be even more pronounced in this population. Additionally, this analysis could have been stronger if there were multiple indicators (i.e., three or more measures) that were being analyzed for each construct (i.e., child mental health, parent mental health, child sleep, parent sleep), which would have enhanced the reliability and robustness of these findings. Furthermore, the relationships between COVID-19 stress and child and parent mental health were not fully explained by child and parent sleep. Variables such as hopelessness, lack of social support, and family conflict may have played a role in the results.

While the large R^2^ values suggest that the model explains a significant portion of variance in mental health outcomes, it is possible that other unmeasured factors, such as socio-economic status, other (non-COVID-19-related) environmental stressors, or pre-existing health conditions may have contributed to these outcomes. Lastly, all measures were reported by parents (i.e., COVID-19 stress, child sleep, child mental health), which may have introduced bias or limitations in capturing the full scope of stress experienced within the household [68].

### 4.3. Implications

Clinicians should be aware of the adverse effects of heightened stress on both child and parent sleep and mental health. The findings of this study underscore the interrelated nature of these issues, highlighting that child and parent sleep problems can significantly exacerbate family mental health difficulties. Therefore, it is essential for health care providers to assess sleep quality and duration alongside mental health, especially in families experiencing heightened stress, as the treatment of sleep problems may reduce the impact of stress on mental health.

Though emergency measures from the COVID-19 pandemic have ended, wait times to see physicians and other clinicians remain high. The average wait time to see a physician in Canada is at an average of 27.7 weeks; wait times in the province of Ontario are ~22 weeks, and waitlists in Nova Scotia are up to 57 weeks [69]. Given the observed effects of parent and child sleep on stress and mental health during the COVID-19 pandemic, online intervention programs targeting both child sleep and mental health could be beneficial. Online programs are cost-effective, easily accessible, and scalable, especially during pandemics when in-person contact is diminished.

### 4.4. Future Directions

As the COVID-19 pandemic has ended, future research should focus on assessing the long-term effects of pandemic-related stress on both parent and child sleep and mental health. While the current study provides valuable insights into immediate impacts, a longitudinal approach could offer a deeper understanding of these effects over time. Though the current study sample had to be living in an area where COVID-19 emergency measures (e.g., lockdowns) were or were not occurring in the period of interest, no comparison was performed in the present study between children who were being home-schooled/going through virtual learning and children who were still attending in-person learning. COVID-19 stress, child and parent sleep, and child and parent mental health may differ between these two groups, as children attending in-person learning may have had increased stress during this time and lower sleep duration. Furthermore, having children self-report on their mental health symptoms, sleep, and stress regarding the COVID-19 pandemic in addition to parent report would help researchers understand children’s perspectives and allow for the assessment of the convergence or divergence of the results across reporting sources. Moreover, intervention studies focusing on mitigating the effects of pandemic-related stress are also needed. For instance, Better Nights, Better Days for COVID-19 has been developed to help parents/caregivers improve sleep in school-aged children [6]. Given the strong relationship between sleep and mental health found in this study, developing and testing sleep-focused interventions that target both parents and children could help alleviate ongoing stress and potentially prevent or reduce mental health difficulties.

Finally, it may be valuable to explore broader societal and environmental impacts on sleep and mental health during the COVID-19 pandemic. For example, future research could investigate how factors such as work-from-home policies, changes in social interaction, and public health messaging may have influenced family dynamics, stress, sleep, and mental health in children and parents. Understanding these broader contexts can help policymakers design better support systems for families in the event of future global crises.

## 5. Conclusions

In summary, the current study found that COVID-19 stress significantly impacted both parent and child mental health, with these effects partially mediated by child and parent sleep problems. For children, their own sleep was a better predictor of their own mental health outcomes, while for parents, both their own sleep and their child’s sleep equally predicted their mental health outcomes. This was observed in a sample of parents from Canada and the US who experienced various levels of emergency measures and restrictions during the COVID-19 pandemic. These findings underscore the need for targeted interventions that address sleep disturbances within families as part of a broader approach to mitigating the mental health impacts of the pandemic. Clinicians and policymakers must prioritize sleep as a key component of mental health care, particularly in the context of long-term COVID-19 pandemic recovery and similar future crises.

## Figures and Tables

**Table 1:** Parental demographics (*N* = 961).

Variable	
Mean age (*SD*)	38.21 (6.61)
Gender	
Man	483 (50.26%)
Woman	475 (49.43%)
Non-Binary	2 (0.21%)
Prefer not to answer	1 (0.10%)
Parent ethnicity	
White	707 (73.60%)
Asian or Arab/West Asian (e.g., Armenian, Egyptian, Iranian, Lebanese, Moroccan) ^a^	145 (15.09%)
Latin American or Black or Indigenous a	68 (7.07%)
Multiracial	32 (3.30%)
Other	4 (0.42%)
Prefer not to answer	5 (0.52%)
Highest level of education completed	
College or university graduate ^b^	726 (75.55%)
Less than college or university completion ^c^	235 (24.45%)
Employment status (15 January–15 February)	
Employed ^d^	770 (80.13%)
Unemployed ^e^	191 (19.87%)
Mean number of children living at-home (*SD*)	1.89 (0.89)
Family income	
CAD 25,000 or less per year	34 (3.54%)
Between CAD 26,000 and 50,000	113 (11.76%)
Between CAD 51,000 and 75,000	184 (19.15%)
Between CAD 76,000 and 100,000	203 (21.12%)
Between CAD 101,000 and 125,000	131 (13.63%)
Between CAD 126,000 and 150,000	127 (13.22%)
CAD 151,000 or more per year	138 (14.36%)
Prefer not to answer	31 (3.22%)
Location	
Canada	805 (83.77%)
Atlantic Provinces ^f^	88 (10.93%)
Central Canada ^g^	477 (59.26%)
Prairie Provinces ^h^	169 (20.99%)
West Coast ^i^	67 (8.32%)
Northern Territories	2 (0.25%)
Unspecified	2 (0.25%)
United States	156 (16.23%)
North-East ^a^	24 (15.38%)
South ^a^	41 (26.28%)
Mid-West ^a^	8 (5.13%)
West ^a^	83 (53.21%)

*Notes.* Canadian provinces and American states were separated into main regions [49,50]. ^a^ Combined to maintain confidentiality of respondents due to low numbers in one or more of these categories. ^b^ Includes participants who indicated being a college/university graduate, having completed some post-graduate education or having a post-graduate degree. ^c^ Includes participants who indicated that they completed elementary school, some high school, are a high school graduate or completed some college/university. ^d^ Includes participants who indicated being employed full or part-time. ^e^ Includes participants who indicated being unemployed, not in the labour force, or a full- or part-time student. ^f^ Includes Nova Scotia, New Brunswick, Prince Edward Island, and Newfoundland and Labrador. ^g^ Includes Ontario and Quebec. ^h^ Includes Manitoba and Saskatchewan. ^i^ Includes Alberta and British Columbia.

**Table 2:** Child demographics (*N* = 961).

Variable	
Mean child age in years (*SD*)	8.64 (2.63)
Child gender	
Boy	551 (57.3%)
Girl	408 (42.46%)
Non-binary	1 (0.10%)
Prefer not to answer	1 (0.10%)
Child ethnicity	
White	687 (71.49%)
Asian or Arab/West Asian (e.g., Armenian, Egyptian, Iranian, Lebanese, Moroccan) ^a^	127 (13.21%)
Latin America or Black or Indigenous ^a^	63 (6.55%)
Other	12 (1.25%)
Prefer not to answer	9 (0.94%)
Multiracial	63 (6.56%)

^a^ Combined to maintain confidentiality of respondents due to low numbers in one or more of these categories.

**Table 3:** Bivariate correlations, means, standard deviations, and ranges.

Measure	1	2	3	4	5
1. COVID-19 stress	-				
2. Child sleep problems	0.45 ***	-			
3. Parent sleep problems	0.21 ***	0.38 ***	-		
4. Child mental health difficulties	0.51 ***	0.64 ***	0.39 ***	-	
5. Parent mental health difficulties	0.40 ***	0.52 ***	0.51 ***	0.58 ***	-
Mean	3.80	12.75	10.87	9.03	10.65
Standard deviation	2.55	7.34	9.34	3.36	3.63
Possible range	0–8	4–20	4–20	0–40	0–42
Observed range	0–7	0–31	0–42	4–19	4–20

*Notes*. *** *p* < 0.001.

## Data Availability

The datasets generated and analyzed during the current study are not publicly available due to keeping participant’s identity and information confidential. The researchers do not have ethical approval to share the data.

## Abbreviations

The following abbreviations are used in this manuscript:

COVID-19	Coronavirus Disease of 2019
